# Two-dose-level confirmatory study of the pharmacokinetics and tolerability of everolimus in Chinese patients with advanced solid tumors

**DOI:** 10.1186/1756-8722-4-3

**Published:** 2011-01-13

**Authors:** BingHe Xu, YiLong Wu, Lin Shen, DingWei Ye, Annette Jappe, Azzeddine Cherfi, Hui Wang, RuiRong Yuan

**Affiliations:** 1 Cancer Hospital Chinese Academy of Medical Sciences, Beijing, China; 2 Guandong General Hospital, Guandong, China; 3 Beijing Cancer Hospital, Beijing, China; 4 Cancer Hospital Fundan University, Shanghai, China; 5 Novartis Pharma AG, Basel, Switzerland; 6 Novartis Pharma Co., Ltd., Beijing, China; 7 Novartis Pharmaceuticals Corporation, Florham Park, NJ, USA; New Jersey Medical School (UMDNJ), Newark, NJ, USA

## Abstract

**Background:**

This phase I, randomized, multicenter, open-label study investigated the pharmacokinetics, safety, and efficacy of the oral mammalian target of rapamycin inhibitor everolimus in Chinese patients with advanced solid tumors.

**Methods:**

A total of 24 patients with advanced breast cancer (n = 6), gastric cancer (n = 6), non-small cell lung cancer (n = 6), or renal cell carcinoma (n = 6) who were refractory to/unsuitable for standard therapy were randomized 1:1 to oral everolimus 5 or 10 mg/day. Primary end points were pharmacokinetic parameters and safety and tolerability. Pharmacokinetic 24-h profiles were measured on day 15; trough level was measured on days 2, 8, 15, 16, and 22. Tolerability was assessed continuously. This final analysis was performed after all patients had received 6 months of study drug or had discontinued.

**Results:**

Everolimus was absorbed rapidly; median T_max _was 3 h (range, 1-4) and 2 h (range, 0.9-6) in the 5 and 10 mg/day groups, respectively. Pharmacokinetic parameters increased dose proportionally from the 5 and 10 mg/day doses. Steady-state levels were achieved by day 8 or earlier. The most common adverse events suspected to be related to everolimus therapy were increased blood glucose (16.7% and 41.7%) and fatigue (16.7% and 33.3%) in the everolimus 5 and 10 mg/day dose cohorts, respectively. Best tumor response was stable disease in 10 (83%) and 6 (50%) patients in the 5 and 10 mg/day groups, respectively.

**Conclusions:**

Everolimus 5 or 10 mg/day was well tolerated in Chinese patients with advanced solid tumors. The observed safety and pharmacokinetic profile of everolimus from this study were consistent with previous studies.

**Trial registration:**

Chinese Health Authorities 2008L09346

## Background

The mammalian target of rapamycin (mTOR), a highly conserved serine-threonine kinase, is a central regulator of critical cell processes via the PI3K-AKT pathway. mTOR signaling is mediated through phosphorylation of downstream substrates p70 ribosomal S6 kinase 1 and eukaryotic initiation factor 4E binding protein 1 resulting in increased translation of proteins promoting cell proliferation and cellular metabolism [1,2]. mTOR also promotes angiogenesis via enhanced hypoxia-inducible factor-1 and growth factor protein translation and increased endothelial and smooth muscle cell proliferation [3,4]. The PI3K/AKT/mTOR-signalling pathway has been shown to be dysregulated in a variety of human malignancies [5-8], making mTOR inhibition a rationale in anticancer therapy.

Everolimus, an orally available mTOR inhibitor, binds to immunophilin FK506-binding protein 12 to inhibit mTOR activity [4,9]. Everolimus is approved currently in the United States, Europe, and Japan for the treatment of patients with metastatic renal cell carcinoma (RCC) whose disease has progressed on sunitinib or sorafenib [10]. The pivotal phase III study of everolimus 10 mg daily demonstrated significantly prolonged progression-free survival compared with placebo in this patient population [11]. Everolimus was generally well tolerated, with most adverse events mild or moderate in severity [11].

Preclinical studies have shown that everolimus inhibits proliferation of a wide spectrum of human solid tumors *in vitro *and *in vivo *[12-14]. Pharmacokinetic (PK) studies of everolimus in patients with advanced solid tumors have shown that absorption of everolimus is rapid and that PK parameters at steady state, including exposure and maximum and minimum plasma concentrations, exhibit dose-proportional responses over a dose range of 5 to 10 mg/day [9,10,15]. These doses have been demonstrated to provide effective inhibition of mTOR activity and encouraging antitumor activity in patients with advanced solid tumors, including breast, lung, colorectal, renal, ovarian, and prostate cancers [9,15,16].

The PK profiles of daily everolimus have been investigated in Japanese and predominantly white cancer patients from the United States and Europe and were found to be similar [9,17]. However, no data are available currently in Chinese patients. Based on the preclinical and global safety and efficacy data, everolimus may provide similar clinical benefit to Chinese patients with advanced solid tumors. This phase I study was recommended by the China State Food and Drug Administration to evaluate PK, safety, and antitumor activity of oral everolimus 5 and 10 mg/day in Chinese patients with advanced solid tumors in part to support global phase III studies to be conducted in China.

## Methods

### Patients

Eligible patients were aged ≥18 years with a histologically confirmed diagnosis of advanced breast cancer, gastric cancer, non-small cell lung cancer (NSCLC), or RCC and were unsuitable for standard anticancer therapy because of treatment-refractory disease or other reasons. These malignancies were selected as inclusion criteria because they are the most common cancers among the Chinese population [18] and have been shown to respond to everolimus in non-Chinese patient populations with advanced breast cancer, gastric cancer, NSCLC, or RCC [11,19-22]. Patients had to have ≥1 measurable lesion as defined by Response Evaluation Criteria in Solid Tumors (RECIST) [23]; adequate bone marrow, liver, and renal functions; controlled diabetes (fasting serum glucose ≤1.5 × upper limit of normal); a body weight ≥50 kg and ≤100 kg with a body mass index ≤32 kg/m2; and a World Health Organization performance status of 0-2. Exclusion criteria included primary central nervous system tumors or metastases, uncontrolled infection, seropositive for human immunodeficiency virus or hepatitis B/C, gastrointestinal impairment or disease that could significantly alter the absorption of everolimus, antineoplastic therapy within 30 days (6 weeks for nitrosoureas or mitomycin-C), radiation therapy within 4 weeks, surgery within 3 weeks before starting study drug, or treatment with strong CYP3A inhibitors or inducers within 5 days before starting study drug. All patients gave written informed consent before study entry according to the Good Clinical Practice guidelines of the International Conference on Harmonization and national regulations. The protocol was reviewed and approved by the ethics committee at each participating institution.

### Study Design

**Figure 1:**
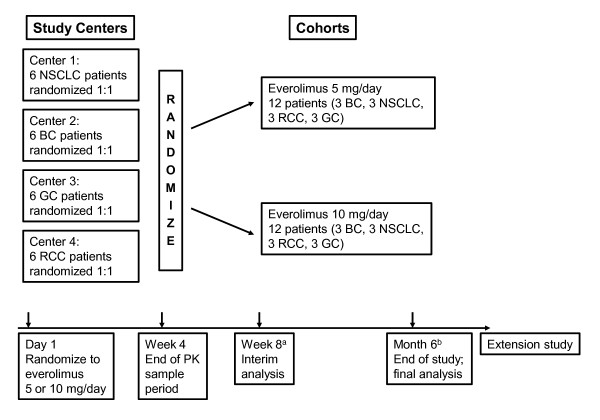
**Study schema**. ^a^At interim analysis, all patients had received 8 weeks of treatment or discontinued before week 8. ^b^The final analysis was performed after all patients had received 6 months of study treatment or discontinued treatment before month 6. BC = breast cancer; GC = gastric cancer; NSCLC = non-small cell lung cancer; PK = pharmacokinetic; RCC = renal cell carcinoma.

In this randomized, open-label, phase I study conducted in 4 clinical centers in China, patients with advanced cancer were randomized 1:1 to receive everolimus 5 mg/day or 10 mg/day (Figure 1). Dose modifications were permitted when patients could not tolerate the protocol-specified dosing schedule. In the event of everolimus-suspected toxicity, the investigator was to follow the study drug modification/interruption guidelines. A patient was kept at the initial dose level (10 mg daily or 5 mg daily) when the toxicity was tolerable. However, if toxicity became intolerable, the study drug was interrupted until recovery to grade ≤1 and then re-introduced at the initial dose or at a lower dose level (reduced to 5 mg daily for the 10 mg/day cohort, or 5 mg every other day for the 5 mg/day cohort) depending on the type of toxicity and its severity. All study drug interruptions or dose modifications were to be documented on the case report/record form. Study drug was provided by Novartis Oncology (Florham Park, NJ), the trial sponsor. Randomization was stratified by center and cancer type, with each center representing 1 cancer type.

Patients continued treatment until tumor progression, unacceptable toxicity, death, or discontinued if the investigator or patient felt it was in the patient's best interest to discontinue participation. Dose modifications were allowed in the event of adverse events grade ≥2. Specific nomograms were followed to manage patients who developed known toxicities of everolimus, such as non-infectious pneumonitis.

### Assessments and Analyses

Primary end points were PK parameters and safety and tolerability. The secondary end point was objective response. Evaluations were performed within 2 days before the first dose of everolimus (baseline), weekly for the first 4 weeks, every other week for the second and third month, and monthly thereafter. A safety follow-up was conducted 28 days after the last dose of everolimus.

Blood samples for everolimus 24-h PK profile were collected on day 15 pre-dose and at 1, 2, 4, 6, 8, and 24 h post-dose; blood samples for everolimus trough concentration were collected pre-dose on days 2, 8, 16, and 22. Everolimus concentration was determined after liquid extraction by a liquid chromatography-mass spectroscopy method with lower limits of quantification for everolimus of 0.3 ng/mL. PK parameters of everolimus determined for each cohort included the maximum blood concentration (C_max_), minimum blood concentration (C_min_), time to maximum concentration (T_max_), area under the dosing curve (AUC_0-τ_), and total body apparent clearance of drug from the blood (CL/F). PK analyses were performed on all patients in the safety population with a sufficient number of evaluable blood samples.

Safety assessments included incidence, severity, and treatment relationship of adverse and serious adverse events and the regular monitoring of hematology, serum and urine chemistry, vital signs, and physical condition. Adverse events were graded according to the National Cancer Institute's Common Terminology Criteria for Adverse Events, version 3.0 [22]. The safety population consisted of all patients who received ≥1 dose of study drug and had ≥1 post-baseline safety assessment.

Tumor response and progression was assessed locally for all randomized patients using RECIST criteria. A computed tomography scan or magnetic resonance image of the chest, abdomen, and pelvis was performed at screening and every 2 months (±7 days) thereafter. Confirmatory imaging results ≥4 weeks after an initial observation were required for a positive assessment of complete or partial response.

This final analysis was performed after all patients had received 6 months of study drug or had discontinued from the study.

## Results

### Patients

A total of 27 patients were screened for study participation. Of the 24 Chinese patients (6 patients per tumor type) enrolled in the study, 12 received everolimus 5 mg/day and 12 received everolimus 10 mg/day. Patient demographic and baseline characteristics, including treatment history, were similar between the 2 dose cohorts (Table 1). At the time of data cutoff for the final analysis, 2 patients with RCC in the everolimus 5 mg/day cohort and 1 patient with breast cancer in the everolimus 10 mg/day cohort were still receiving treatment. A total of 10 patients in the everolimus 5 mg/day group and 11 patients in the everolimus 10 mg/day group had discontinued. The most common reason for treatment discontinuation was disease progression (Table 2). All 24 patients were included in the full analysis set and in the safety population.

**Table 1:** Patient characteristics

Characteristic	Everolimus 5 mg/day (n = 12)	Everolimus 10 mg/day (n = 12)
Median (range) age, y	55 (27-75)	56 (32-75)
Sex, n (%)		
Male	5 (41.7)	4 (33.3)
Female	7 (58.3)	8 (66.7)
WHO performance status, *n *(%)		
0	3 (25.0)	1 (8.3)
1	9 (75.0)	9 (75.0)
2	0	2 (16.7)
Prior antineoplastic therapy, n (%)		
Surgery	11 (91.7)	10 (83.3)
Radiotherapy	3 (25.0)	3 (25.0)
Chemotherapy	10 (83.3)	10 (83.3)
Targeted therapy	5 (41.7)	5 (41.7)
Immunotherapy	3 (25.0)	2 (16.7)
Hormonal therapy	2 (16.7)	0

WHO = World Health Organization.

**Table 2:** Patient disposition

	Everolimus 5 mg/day (n = 12)	Everolimus 10 mg/day (n = 12)
Ongoing, n (%)	2 (16.7)	1 (8.3)
Discontinued, n (%)	10 (83.3)	11 (91.7)
Patient withdrew consent	1 (8.3)	2 (16.7)
Death	0	2 (16.7)
Disease progression	9 (75.0)	7 (58.3)

### Treatment Exposure

The median durations of exposure to everolimus were 136.5 days in the 5 mg/day cohort and 63.5 days in the 10 mg/day cohort. The patients with RCC in this study remained on treatment the longest with median duration of exposure of 184.5 (range, 65-130) compared with patients with breast cancer (93.0 days; range, 47-243), gastric cancer (88.5 days; range, 46-247), or NSCLC (73.5 days; range, 42-153). For the 3 patients still participating in the study at the time of data cutoff, everolimus exposures were 209 and 230 days for the 2 patients with RCC and 243 days for the patient with breast cancer.

### Pharmacokinetics

**Figure 2:**
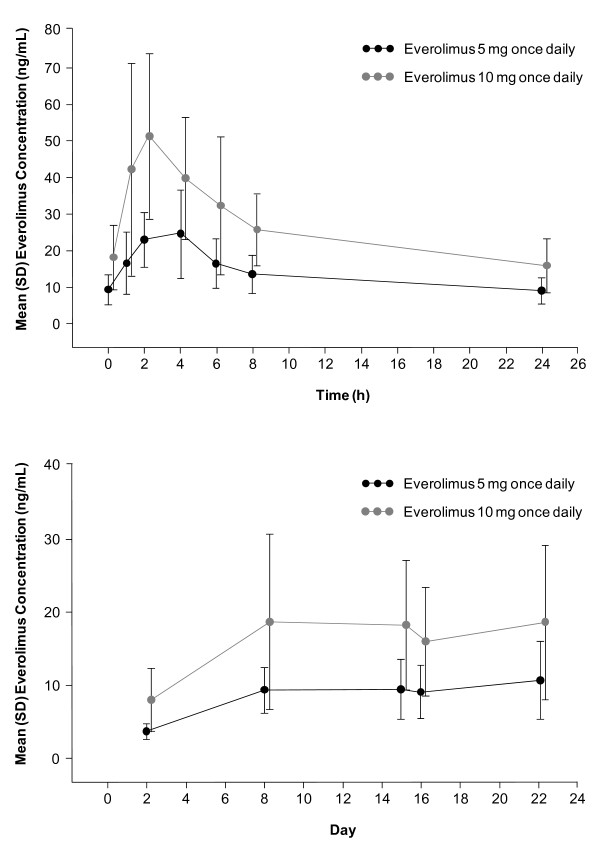
**Everolimus 24-h blood concentration-time profiles on day 15 and everolimus blood trough concentration-time profiles during continuous oral dosing for 28 days**. Error bars indicate standard deviation.

Absorption of everolimus after oral administration was rapid to moderate with a median T_max _of 3.0 h in the 5 mg/day cohort and 2.0 h in the 10 mg/day cohort (Figure 2, Table 3). The values for C_min_, C_max_, and AUC_0-τ _with 10 mg/day were approximately 2-fold those at 5 mg/day and increased dose proportionally. After reaching steady state on day 8, mean (± standard deviation) values of CL/F were 16.7 (±5.6) and 18.2 (±7.2) L/h at doses of 5 and 10 mg/day, respectively (Figure 2). The similarity of CL/F between the 5 mg/day and 10 mg/day dose cohorts supports PK linearity.

**Table 3:** Pharmacokinetic parameters of everolimus

**Parameter**^a^	Everolimus 5 mg/day (n = 12)	Everolimus 10 mg/day (n = 12)
AUC_0-τ_, h•ng/mL	316.1 (34.8)	588.6 (41.4)
CL/F, L/h	15.8 (34.8)	16.99 (41.4)
C_max_, ng/mL	28.8 (31.3)	53.8 (56.2)
T_max_, h (range)	2.96 (1.00-4.00)	2.0 (0.92-6.00)
C_min_, ng/mL	8.3 (46.8)	14.5 (47.9)

AUC_0-τ _= area under the blood concentration-time curve; CL/F = total body apparent clearance of drug from the blood; C_max _= maximum blood concentration; C_min _= pre-dose trough concentration; T_max _= time to reach maximum blood concentration.

^a^Values shown are means (% coefficient of variation) unless otherwise indicated.

### Safety

All 24 patients reported ≥1 adverse event; most were grade 1/2 events that resolved without additional treatment (Table 4). The most common adverse events with a suspected relationship to everolimus in the everolimus 5 mg/day and 10 mg/day dose cohorts were hyperglycemia (16.7% and 41.7%) and fatigue (16.7% and 33.3%, respectively). Three (25%) patients in each dose cohort had grade 3 adverse events suspected to be related to everolimus.

**Table 4:** Adverse events with suspected relationship to everolimus

	Everolimus 5 mg/day (n = 12)			Everolimus 10 mg/day (n = 12)		
Adverse event, n (%)	All grades	Grade 3	Grade 4	All grades	Grade 3	Grade 4
**Total**	**12 (100)**	**3 (25.0)**	**0**	**12 (100)**	**3 (25.0)**	**0**
Hyperglycemia	2 (16.7)	0	0	5 (41.7)	1 (8.3)	0
Fatigue	2 (16.7)	0	0	4 (33.3)	1 (8.3)	0
Anemia	1 (8.3)	1 (8.3)	0	3 (25.0)	0	0
Blood alkaline phosphatase increased	1 (8.3)	0	0	3 (25.0)	1 (8.3)	0
Thrombocytopenia	2 (16.7)	1 (8.3)	0	3 (25.0)	0	0
Hypokalemia	3 (25.0)	1 (8.3)	0	2 (16.7)	0	0
Upper respiratory tract infection	1 (8.3)	1 (8.3)		0	0	0

Three deaths (1 breast cancer and 2 NSCLC patients) occurred during the study; 2 were in the 10 mg/day cohort and 1 was in the 5 mg/day cohort. These events were considered unrelated to everolimus. Underlying cause for all 3 patients was disease progression. One patient with NSCLC in the 10 mg/day cohort experienced venous embolism, which led to aggravated condition and death. Another patient with NSCLC in the everolimus 5 mg/day cohort experienced cerebral hemiplegia related to brain metastases from lung cancer. One patient with breast cancer discontinued study treatment on day 47 due to disease progression and died 2 days later.

### Tumor Response

No complete or partial responses were observed. The best overall tumor response was stable disease for 10 patients (83.3%) in the everolimus 5 mg/day dose cohort and 6 (50.0%) patients in the everolimus 10 mg/day cohort. Median duration of stable disease was 5.03 months (95% confidence interval [CI], 2.89-8.05) for the 5 mg/day dose cohort and 6.08 months (95% CI, 3.58-not reached) for the 10 mg/day dose cohort. Of the patients with stable disease, 3 had breast cancer, 4 had NSCLC, 5 had gastric cancer, and 4 had RCC. Two (16.7%) patients in the 5 mg/day cohort and 5 (41.7%) patients in the 10 mg/day cohort had progressive disease as best overall response. One patient with NSCLC in the 10 mg/day group had a best overall response of unknown (died before first post-baseline tumor assessment).

## Discussion

This phase I study confirms the PK, safety, and efficacy of everolimus 5 or 10 mg/day in a limited population of adult Chinese patients with advanced cancers. These findings are consistent with the results of previous studies in Asian and non-Asian study populations [11,17,19-21].

Absorption of everolimus after oral administration was rapid, with maximum blood concentrations generally reached after 2 to 4 h. PK parameters exhibited a dose-proportional response, and steady-state levels were achieved within 8 days of treatment. The everolimus steady-state area under the concentration-time curve (AUC_0-τ_) and maximum drug concentration (C_max_) is dose-proportional over the 5 mg and 10 mg dose range in the daily regimen. Japanese and white patients with cancer with similar liver functions have similar clearance values. Neither age nor gender has significant effects on PK of everolimus in patients with cancer. The PK profiles of everolimus 5 mg/day and 10 mg/day in this Chinese patient population are similar to those observed previously in white patients from the United States and Europe who had advanced solid tumors [9].

Everolimus doses up to 10 mg/day were well tolerated in adult Chinese patients with advanced solid tumors with hyperglycemia and fatigue as the most commonly reported adverse events suspected to everolimus treatment. The safety profile of everolimus in Chinese patients is consistent with that of previous studies, including the pivotal global phase III metastatic RCC study [9,11,15]. In the phase III study in RCC, the most common everolimus-related adverse events were anemia and metabolic abnormalities, including hyperglycemia and hyperlipidemia, which are considered to be the result of inhibition of mTOR-regulated glucose and lipid metabolism [9,11,15]. Stomatitis, rash, and fatigue also are known class effects of mTOR inhibitors [11,24-26]. Noninfectious pneumonitis, a key adverse event associated with mTOR inhibition [27], was not observed in this study. Grade 3 upper respiratory tract infection was reported in 1 patient, but the condition was reversible with remedial treatment and interruption of everolimus therapy. Three patients died on study due to disease progression. One of the patients experienced cerebral hemiplegia related to brain metastases from lung cancer. None of the deaths was suspected to be related to everolimus treatment.

Although efficacy results are preliminary, clinically, antitumor activity of everolimus in the form of disease stabilization was observed in 16.7% of the patient population across a broad spectrum of malignancies. Efficacy results trended toward support of 5 mg/day dosing; however, the patient population is too small to confirm a meaningful difference between the 2 dose cohorts. The efficacy findings are consistent with the results of previous studies in Asian and non-Asian study populations [19-21]. In particular, disease stabilization observed in 4 of 6 patients with RCC in this study confirmed the efficacy of everolimus in Chinese patients with RCC, consistent with experience from the larger phase III study in RCC [11]. At the time of data cutoff, the median duration of stable disease for the 4 patients with RCC had not yet been reached (95% CI, 6.08-11.1+), and 2 patients with stable RCC remained on treatment with everolimus.

## Conclusions

The results of this phase I study suggest that everolimus 5 or 10 mg/day is safe and well tolerated in Chinese patients with advanced solid tumors. Overall, the results warrant additional clinical evaluation of everolimus 5 to 10 mg/day in this patient population.

## Abbreviations

AUC_0-τ _= area under the blood concentration-time curve; BC = breast cancer; CL/F = total body apparent clearance of drug from the blood; C_max _= maximum blood concentration; C_min _= pre-dose trough concentration; GC = gastric cancer; mTOR = mammalian target of rapamycin; NSCLC = non-small cell lung cancer; PK = pharmacokinetic; RCC = renal cell carcinoma; RECIST = Response Evaluation Criteria in Solid Tumors; T_max _= time to reach maximum blood concentration; WHO = World Health Organization.

## Competing interests

AJ, AC, HW, and RY are employees of and have equity interest in Novartis.

## Authors' contributions

BX and YW participated in the design of the study, carried out parts of the study, wrote the manuscript, and contributed equally to this study; LS and DY carried out parts of the study and contributed to critical review of the manuscript; HW and AJ designed the project, collected data, analyzed data, and contributed to critical review of the manuscript; AC analyzed data and contributed to critical review of the manuscript; RY designed the project, collected data, analyzed data, and wrote the manuscript; all authors read and approved this manuscript.
